# Molecular cloning and characterization of a flavonoid-*O*-methyltransferase with broad substrate specificity and regioselectivity from *Citrus depressa*

**DOI:** 10.1186/s12870-016-0870-9

**Published:** 2016-08-22

**Authors:** Nobuya Itoh, Chisa Iwata, Hiroshi Toda

**Affiliations:** Biotechnology Research Center and Department of Biotechnology, Toyama Prefectural University, 5180 Kurokawa, Imizu, Toyama 939-0398 Japan

**Keywords:** *O*-methyltransferase, flavonoid, *S*-adenosyl-l-methionine dependent, *Citrus depressa*, nobiletin

## Abstract

**Background:**

Flavonoids are secondary metabolites that play significant roles in plant cells. In particular, polymethoxy flavonoids (PMFs), including nobiletin, have been reported to exhibit various health-supporting properties such as anticancer, anti-inflammatory, and anti-pathogenic properties. However, it is difficult to utilize PMFs for medicinal and dietary use because plant cells contain small amounts of these compounds. Biosynthesis of PMFs in plant cells is carried out by the methylation of hydroxyl groups of flavonoids by *O*-methyltransferases (FOMT), and many kinds of FOMTs with different levels of substrate specificity and regioselectivity are cooperatively involved in this biosynthesis.

**Results:**

In this study, we isolated five genes encoding FOMT (*CdFOMT1*, *3*, *4*, *5*, and *6*) from *Citrus depressa*, which is known to accumulate nobiletin in the peels of its fruits. The genes encoded Mg^2+^-independent *O*-methyltransferases and showed high amino acid sequence similarity (60–95 %) with higher plant flavonoid *O*-methyltransferases. One of these genes is *CdFOMT5*, which was successfully expressed as a soluble homodimer enzyme in *Escherichia coli*. The molecular mass of the recombinant *CdFOMT5* subunit was 42.0 kDa including a 6× histidine tag. The enzyme exhibited *O*-methyltransferase activity for quercetin, naringenin, (-)-epicatechin, and equol using *S*-adenosyl-l-methionine (SAM) as a methyl donor, and its optimal pH and temperature were pH 7.0 and 45 °C, respectively. The recombinant CdFOMT5 demonstrated methylation activity for the 3-, 5-, 6-, and 7-hydroxyl groups of flavones, and 3,3′,5,7-tetra-*O*-methylated quercetin was synthesized from quercetin as a final product of the whole cell reaction system. Thus, CdFOMT5 is a *O*-methyltransferase possessing a broad range of substrate specificity and regioselectivity for flavonoids.

**Conclusions:**

Five FOMT genes were isolated from *C. depressa*, and their nucleotide sequences were determined. CdFOMT5 was successfully expressed in *E. coli* cells, and the enzymatic properties of the recombinant protein were characterized. Recombinant CdFOMT5 indicated *O*-methyltransferase activity for many flavonoids and a broad regioselectivity for quercetin as a substrate. Whole-cell biocatalysis using CdFOMT5 expressed in *E. coli* cells was performed using quercetin as a substrate, and 3,3′,5,7-tetramethylated quercetin was obtained as the final product.

**Electronic supplementary material:**

The online version of this article (doi:10.1186/s12870-016-0870-9) contains supplementary material, which is available to authorized users.

## Background

Flavonoids are major secondary metabolites in plants. More than 10,000 kinds of flavonoid derivatives are estimated to occur in plants [1]. Such varieties of flavonoid subgroups include flavones, flavonols, flavanones, flavanes, flavanols, isoflavones, and anthocyanidins, all of which originate from the phenylpropanoid synthesis pathway [2, 3]. In plant cells, flavonoids are modified by many enzymes, such as methyltransferases, glycosyltransferases, sulfotransferases, acyltransferases, oxidases, and others. These modifications, coupled with the structural variation observed in different flavonoid subgroups, contribute to a huge diversity of flavonoids [4, 5].

It is well known that many flavonoids have significant roles in plants, such as inflorescence pigments and antioxidants as well as serving as substances with anti-pathogenic [6, 7], anti-insect [8], and signaling functions [9]. Recently, it has been reported that *O*-methylated flavonoids have significant biological activity in humans, exhibiting antibiotic [10], antiviral [11], anti-cancer [12, 13], anti-inflammatory [14], anti-obesity [15, 16], neuroprotective [17, 18], and anti-allergy [19] properties. Increasing evidence of the effects of *O*-methylated flavonoids on human health suggests they can be used to enrich processed foods or in dietary supplements and pharmaceuticals. Nobiletin (3′,4′,5,6,7,8-hexamethoxyflavone) is one of the abundant polymethoxy flavonoids (PMF) in *Citrus depressa* peels, and it has been reported to possess several bioactivities [13, 14, 20, 21]. However, it is difficult to investigate nobiletin in general and medical research because of the small amounts of nobiletin obtained from *Citrus* peels. In plant cells, hydroxyl groups of flavonoids are methylated by reactions with *O*-methyltransferase (OMT) using *S*-adenosyl-l-methionine (SAM) as a methyl donor.

Plant OMTs are generally classified as either class I or class II by their structural and enzymatic properties [22]. Class I OMTs, which include the well-known enzyme caffeoyl-CoA 3-OMT (CCoAOMT), are characterized by low subunit molecular masses (from 23 to 27 kDa), dependence on Mg^2+^ ions, and the ability to catalyze the methylation of 3-hydroxyl groups of caffeoyl-CoA to produce feruloyl-CoA. Thus, CCoAOMT, which is involved in lignin biosynthesis in plant cells, is the key enzyme for producing monolignols [23, 24]. Class II OMTs have a higher subunit molecular mass than CCoAOMTs (from 38 to 43 kDa) and do not require Mg^2+^ ions for methylation. Plant cells accumulate many flavonoids through the reactions of such OMTs. Ibrahim et al. reported that cell-free extracts of *Citrus mitis* exhibited stepwise *O*-methylation activities for various flavonoids and suggested that different types of OMTs are involved in the biosynthesis of PMFs in some tissues (peel, root, and callus tissue) [25, 26]. More recently, it was reported that there are 58 OMT genes in the *C. sinensis* (sweet orange) genome, and these genes show distinct expression patterns that differ among tissues and developmental stages [27]. These findings strongly suggested that the structural diversity of PMFs in citrus is caused by combinations of various types of substrate- and regio-specific methyltransferases.

Here, we report the isolation and characterization of five flavonoid *O*-methyltransferase (FOMT) genes (*CdFOMT1*, *3*, *4*, *5*, and *6*) from *C. depressa*. Furthermore, CdFOMT5 was successfully expressed in *E. coli* as a functional enzyme, and its properties were characterized in detail. CdFOMT5 possessed methyltransferase activity for quercetin, a ubiquitous flavonoid in plants, and exhibited a broad range of substrate specificity and regioselectivity toward 3-, 5-, 6-, and 7-hydroxyl groups of flavones. Using the biotransformation of quercetin in a CdFOMT5-expressing *E. coli* biocatalyst, we successfully obtained 3,3′,5,7-tetra-*O*-methylated quercetin as a final product, suggesting that the enzyme participates in the biosynthesis of nobiletin.

## Results and discussion

### Cloning of *CdFOMT* genes from *C. depressa*

To isolate FOMT-coding genes from *C. depressa*, we performed degenerate PCR using primers designed from the conserved amino acid sequences of higher plant FOMTs. Using genomic DNA as the template, we obtained five fragments, whose deduced amino acid sequences showed similarity to several higher plant FOMTs. In order to obtain the full-length *FOMT* genes from *C. depressa*, we performed thermal asymmetric interlaced PCR (TAIL-PCR) and obtained five FOMT-coding genes, *CdFOMT1*, *3*, *4*, 5 and 6. All primers used to clone *CdFOMT* genes are listed in Additional file 1: Table S1. Then, we amplified full-length cDNA of these *CdFOMT* genes using specific primers designed from the deduced N-terminal and C-terminal sequences. Thus, we isolated five *FOMT* genes which encode homologous proteins CdFOMT1, 3, 4, 5, and 6. The amino acid sequences of CdFOMT3, 5, and 6 showed relatively high identities each other (from 67.8 to 82.3 %), although those of CdFOMT1 and 4 showed lower identities (from 28.9 to 39.9 %) with other CdFOMTs (Table 1).

**Table 1:** Comparative identities (%) of amino acid sequences of five FOMTs from *Citrus depressa*

	CdFOMT1	CdFOMT3	CdFOMT4	CdFOMT5	CdFOMT6
CdFOMT1	-	-	-	-	-
CdFOMT3	31.5	-	-	-	-
CdFOMT4	39.9	33.7	-	-	-
CdFOMT5	30.2	67.8	34.7	-	-
CdFOMT6	28.9	82.3	36.1	71.0	-

A comparison of the genomic and cDNA sequences revealed that these *FOMT* genes contain one or three introns (Additional file 2: Figure S1). All *CdFOMT* genes contain an intron at the same position within their coding sequences [Additional file 2: Figure S1, marked with an asterisk; AIXXK-(intron)-(S/W)(I/V)LHDW], although these *CdFOMT* genes show quite low similarities across their whole nucleotide sequences, and the proteins differed substantially in size. Schroder et al. [28] found that *COMT* genes from *Catharanthus roseus* share an intron insertion location. Similarly, the amino acid sequences at this site in *CdFOMT* genes were highly conserved across many plant OMTs. *CdFOMT3* and *CdFOMT5* each contained three introns, which also share positions within their amino acid sequences [QDKXXLXS-(intron1)-WSXLK ~ PHVIXHXPXXP-(intron2)-XXXHVGGDM ~ DAIXXK-(intron3)-(W/S)(I/V)XLHDW], though there were again large differences in both their sizes and sequences as proteins. This result suggests that these *CdFOMT* genes have the same origin and acquired their introns during their shared evolutionary history.

**Fig. 1:**
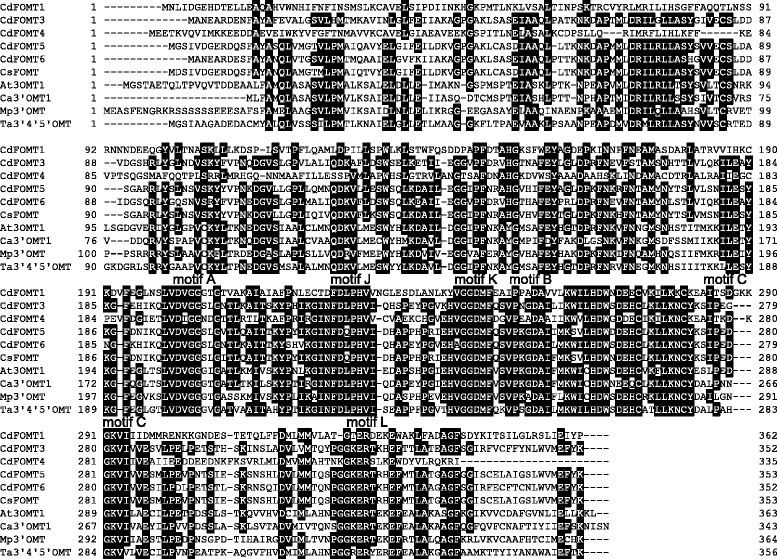
Multiple sequence alignment of *CdFOMT* sequences with higher plant flavonoid *O*-methyl transferases (FOMTs): *C. depressa* (CdFOMT1, DDBJ accession number LC126056; CdFOMT3, LC126057; CdFOMT4, LC126058; CdFOMT5, LC126059; and CdFOMT6, LC126060), *Citrus sinensis* (CsFOMT, GenBank accession number ABP94018), *Arabidopsis thaliana* (At3OMT, AED96460), *Chrysosplenium americanum* (Ca3′OMT1, AAA80579), *Mentha* × *piperita* (Mp3′OMT, AAR09601), and *Triticum aestivum* (Ta3′4′5′OMT, ABB03907). Amino acid sequences were aligned using ClustalW. Black highlighting denotes amino acids that are conserved across many FOMTs

The five newly isolated *CdFOMT* genes from *C. depressa* encode 335 to 362 amino acid residues and shared 60–97 % amino acid sequence identities to known higher plant OMTs (Table 2). Figure 1 shows the alignment of the deduced CdFOMT amino acid sequences and higher plant FOMTs. *CdFOMTs* exhibited several conserved sequences (motifs A, B, C, J, K, and L in Fig. 1), which are likely involved in interactions with the cofactor SAM [22, 29]. The existence of these conserved regions suggests that the five OMT genes obtained from *C. depressa* code for potential SAM-dependent FOMTs.

**Table 2:** Homologs of the *CdFOMT* genes from *Citrus depressa* in the databases

Strain	Homologous gene	Accession No.	Identity (%)
CdFOMT1			
*Citrus sinensis*	predicted trans-resveratrol di-*O*-methyltransferase-like	XP_006480453	87.8
*Fragaria vesca* subsp. *vesca*	predicted *trans*-resveratrol di-*O*-methyltransferase-like	XP_004303127	71.7
*Rosa hybrid* cultivar	orcinol *O*-methyltransferase	AAM23005	71.2
CdFOMT3			
*Citrus sinensis*	predicted caffeic acid 3-*O*-methyltransferase 1-like	XP_006478221	97.1
*Populus trichocarpa*	eugenol *O*-methyltransferase family protein	EEE98552	68.0
*Ricinus communis*	*O*-methyltransferase putative	XP_002515087	63.4
CdFOMT4			
*Vitis vinifera*	*O*-methyltransferase (methoxypyrazine biosynthesis)	AGK93043	67.5
*Medicago truncatula*	flavonoid *O*-methyltransferase-like protein	AES71869	65.7
*Gossypium raimondii*	predicted (*RS*)-norcoclaurine 6-*O*-methyltransferase	KJB47723	64.8
CdFOMT5			
*Citrus sinensis*	predicted caffeic acid 3-*O*-methyltransferase	XP_006494578	97.4
*Populus trichocarpa*	eugenol *O*-methyltransferase family protein	EEE98552	65.0
*Gossypium hirsutum*	caffeic acid 3-*O*-methyltransferase 3	ACZ06242	58.4
CdFOMT6			
*Citrus sinensis*	predicted caffeic acid 3-*O*-methyltransferase-like	XP_006478222	86.3
*Populus euphratica*	predicted caffeic acid 3-*O*-methyltransferase-like	XP_011039447	70.2
*Theobroma cacao*	caffeic acid 3-*O*-methyltransferase 1 isoform 1	EOY32732	68.2

**Fig. 2:**
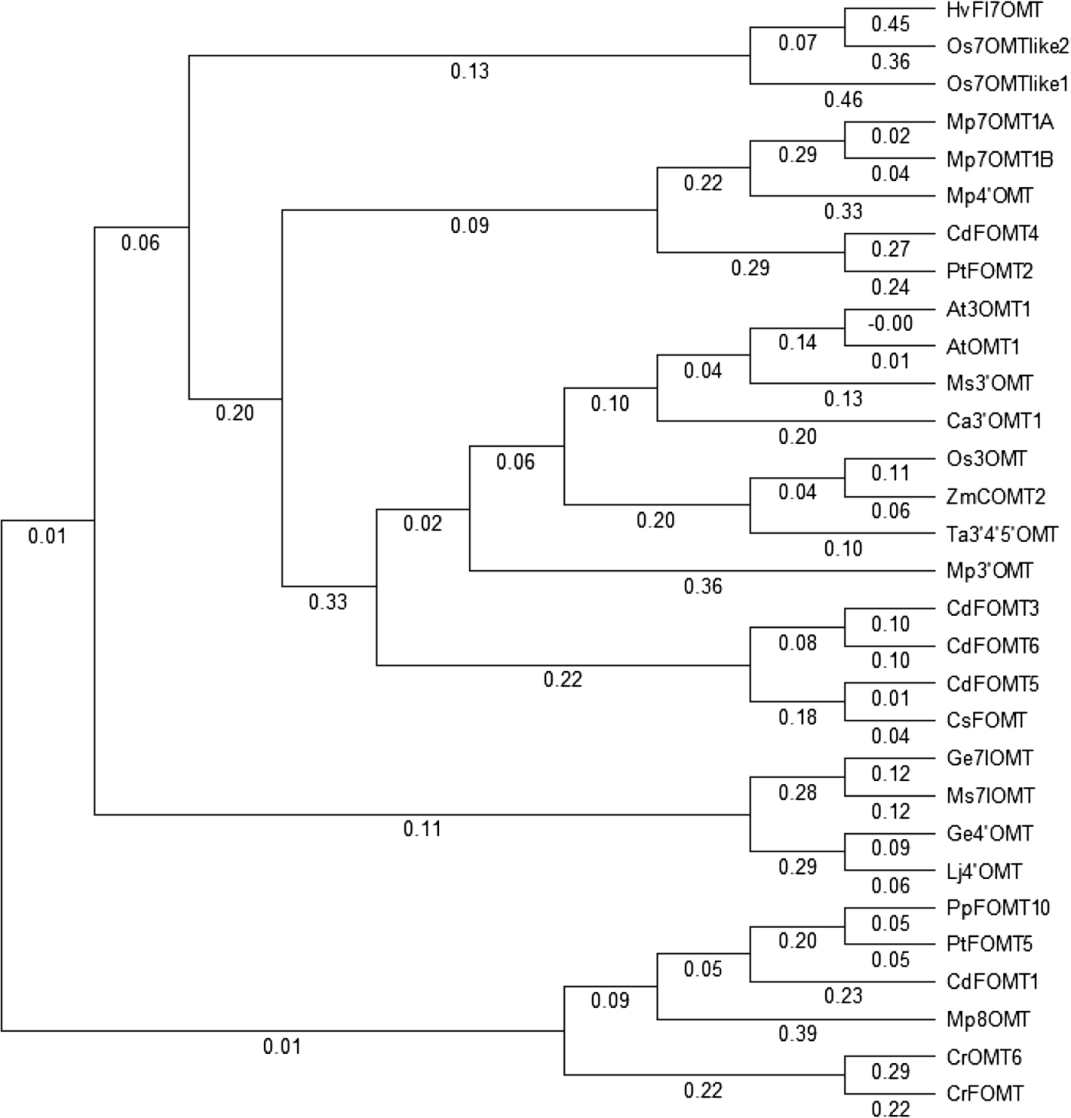
A phylogenetic tree constructed from plant FOMT amino acid sequences. In addition to sequences of CdFOMTs characterized in this paper, 25 plant class II FOMT sequences were also selected using BLAST search and aligned by ClustalW. The phylogenetic tree was constructed using the NJplot program. *Arabidopsis thaliana* (At3OMT, GenBank accession number AED96460 and AtOMT1, AAB9679), *Catharanthus roseus* (CrFOMT, AAM97497 and CrOMT6, AAR02419), *Chrysosplenium americanum* (Ca3′OMT1, AAA80579), *Citrus sinensis* (CsFOMT, ABP94018), *Glycyrrhiza echinata* (Ge4′OMT, AB091684 and Ge7IOMT, AB091685), *Hordeum vulgare* (HvFI7OMT, CAA54616), *Lotus japonicus* (Lj4′OMT, AB091686), *Medicago sativa* (Ms3′OMT, AAB46623 and Ms7IOMT, MSU97125), *Mentha* × *piperita* (Mp3′OMT, AAR09601; Mp4′OMT, AAR09602; Mp7OMT1A, AAR09598; Mp7OMT1B, AAR09599; and Mp8OMT, AAR09600), *Oryza sativa* (Os7OMTlike1, BAD29452; Os7OMTlike2, BAD05699; and Os3OMT, BAF22945), *Populus trichocarpa* (PtFOMT2, EEE86889; PtOMT5, EPR47487; and PtFOMT10, EPR54183), *Triticum aestivum* (Ta3′4′5′OMT, ABB03907), and *Zea mays* (ZmCOMT, ABQ58826)

Phylogenetic analysis of five *CdFOMTs* with 25 putative and defined plant class II OMTs from higher plants (Fig. 2) indicate that they are plant class II OMTs (divalent cation independent). Class II OMTs are generally known to show activity with flavonoids and isoflavonoids [30], while class I OMTs catalyze methylation of phenolic compounds involved in lignin synthesis [23, 24]. These results suggest that the isolated *CdFOMTs* may be involved in the biosynthesis of PMFs such as nobiletin in *C. depressa*.

### Expression of recombinant *CdFOMTs* genes in *Escherichia coli*

**Fig. 3:**
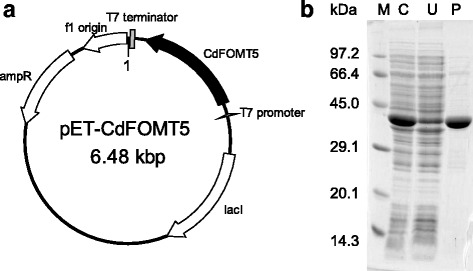
**a** CdFOMT5 expression plasmid and **b** SDS-PAGE analysis of recombinant CdFOMT5 in *E. coli* transformant cells. Lane (M) contained the molecular weight markers; lane (C) the cell-free extract; lane (U) non-adsorbed fraction of Ni-Sepharose column chromatography; and lane (P) the purified enzyme

In order to obtain recombinant enzymes, each *CdFOMT* gene was cloned into a pET21b vector, and *E. coli* BL21(DE3) cells were transformed with a constructed plasmid. With the exception of the CdFOMT5, the four remaining CdFOMTs formed inclusion bodies under several culture conditions and no activity could be detected for these proteins. Schroder et al. [28] reported that three OMT genes in *Catharanthus roseus* (*CrOMT5*, *6*, and *7*) were tested for expression in *E. coli*, and recombinant *CrOMT6* and *CrOMT7* formed soluble proteins whereas *CrOMT5* formed an insoluble protein. In general, expression of functional plant enzymes in *E. coli* is unpredictable and must be determined empirically with many factors such as amino acid composition, protein folding, and post translational modifications influencing the outcome. Thus, the newly isolated methyltransferase genes other than *CdFOMT5* may contribute to biosynthesis of PMF in *C. depressa*, but details of their biochemical functions remain unclear. We were able to successfully express recombinant CdFOMT5 in *E. coli* and confirm its OMT activity. Recombinant CdFOMT5 was obtained as a fusion protein with a 6× histidine tag at the C-terminus and purified by Ni-Sepharose resin column chromatography. Recombinant CdFOMT5 was purified to homogeneity, and a single 42.0-kDa protein band was obtained by an SDS-PAGE analysis (Fig. 3).

### Physicochemical properties of CdFOMT5

Using HPLC, the molecular mass of recombinant CdFOMT5 was estimated to be 88.0 kDa. The theoretical molecular mass of the recombinant CdFOMT5 including the 6× histidine tag is 42.03 kDa, which agrees with the observed molecular mass of 42.0 kDa inferred using SDS-PAGE. It is generally known that typical plant FOMT subunits are homodimers [31, 32]. These findings suggest that recombinant CdFOMT5 is a homodimer protein in *E. coli* cells.

The *pI* value of the enzyme without the 6× histidine tag based on its amino acid sequence was theoretically calculated to be 5.79.

The effects of pH and temperature on OMT activity were measured using quercetin as a substrate (Additional file 2: Figure S2A). Recombinant CdFOMT5 showed optimum activity at pH 7.0 (in potassium phosphate buffer), and its activity fell more than 50 % at pH 5.5 (in Na-citrate buffer) or pH 9.0 (in Tris-HCl buffer; Additional file 2: Figure S2A). The optimum temperature of CdFOMT5 was 45 °C, and the enzyme exhibited more than 80 % of maximum activity at 55 °C (Additional file 2: Figure S2B).

### Regioselectivity of CdFOMT5 for quercetin

**Fig. 4:**
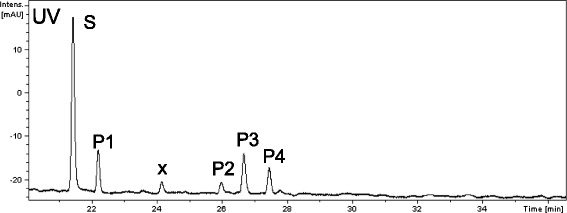
HPLC analysis of quercetin products methylated by CdFOMT5: S, quercetin; P1, 3-*O*-methylated quercetin; P2, azaleatin/rhamnetin; P3, dimethylated quercetin; and P4, trimethylated quercetin. The peak labeled with ‘x’ was not identified as corresponding to flavonoid derivatives. (See Additional file 2: Figure S2)

When *O*-methyltransferase activity was measured using quercetin as a substrate, several peaks corresponding to *O*-methylated products were detected by HPLC analysis (Fig. 4). The retention time for P1 was consistent with 3-*O*-methylquercetin, and that of P2 was consistent with azaleatin (5-*O*-methylquercetin) or rhamnetin (7-*O*-methylquercetin). However, P3 and P4 had retention times that differed from those of quercetin mono-methylated derivatives. To investigate the molecular mass of these compounds, we performed an LC-MS analysis and observed increases of 28- and 42-Da in the molecular ion peaks for P3 and P4, respectively (Additional file 2: Figure S3). This demonstrates that CdFOMT5 can catalyze the *O*-methylation of at least three hydroxyl groups of quercetin and that di- or tri-*O*-methylated quercetin products were obtained by this enzymatic reaction.

**Fig. 5:**
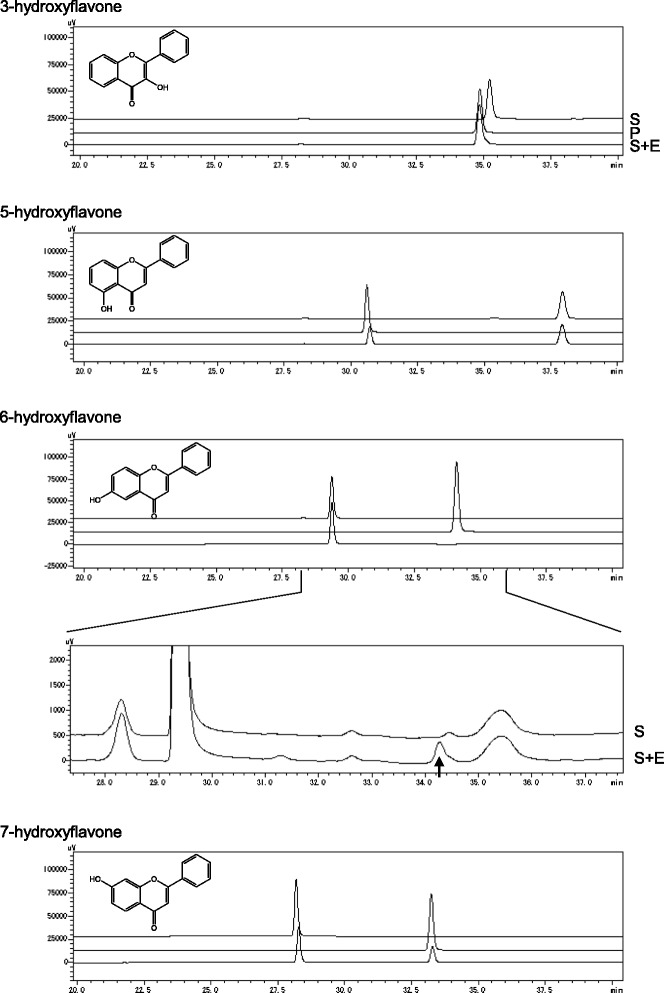
HPLC chromatograms of monohydroxy flavone products methylated by CdFOMT5: S, substrate control; P, authentic compound of methylated product; and S + E, enzyme reaction product

To confirm the regioselectivity of CdFOMT5, the enzymatic reaction was measured using mono-hydroxyflavones as a substrate. As shown in Fig. 5, CdFOMT5 demonstrated methylation activity for 3-, 5-, 6-, and 7-hydroxy groups of flavone. Under the standard assay condition (100 μM substrate), the highest activity was observed for 3-hydroxyflavone (flavonol) based on the peak area of the product, followed by 7-hydroxyflavone with a relative activity of 15.6 % of that of 3-hydroxyflavone and 5-hydroxyflavone with that of 13.5 %. Very weak activity was observed for 6-hydroxyflavone (2 % in comparison with the activity of 3-hydroxyflavone), and no activity was detected for 3′- or 4′-hydroxyflavone and 7-methoxy-8-hydroxyflavone (data not shown). This appears to be the first report of an OMT with the ability to catalyze the *O*-methylation of four positions of hydroxyl groups in the A and C rings of flavonoids. However, there are some reports of sequential methylation of flavonoids by OMTs [33, 34]. In particular, TaOMT2 from wheat exhibited definite sequential methylation of tricetin at its B-ring 3′-, 5′-, and 4′-hydroxyl groups. However, in the CdFOMT5 reaction, not only 3-*O*-methylated quercetin but also azaleatin (5-*O*-methylated quercetin)/rhamnetin (7-*O*-methylated quercetin) were obtained from quercetin, and this result indicates that CdFOMT5 does not exhibit a sequential methylation order for 3-, 5-, and 7-hydroxyl groups of quercetin.

Nobiletin is a major PMF in *C. depressa* peel, and its 3′-, 4′-, 5-, 6-, 7-, and 8-hydroxy groups are all methylated. Therefore, CdFOMT5 is hypothesized to play a significant role in the biosynthesis of PMF in *C. depressa*. There are many reports that a combination of several OMTs catalyze the *O*-methylation of flavonoids to produce PMFs [35, 36]. Furthermore, Joe et al. [37] reported that mutant OMT (POMT7-M1) exhibits methylation activity for 3- and 7-hydroxy groups of flavonoids, whereas native POMT7 can methylate only the 7-hydroxy group [37]. They also showed that substitution of Asp with Gly at amino acid position 257 significantly affected the regioselectivity of POMT7. Although CdFOMT5 contains Asp260 corresponding to the Asp257 of native POMT7, CdFOMT5 was able to catalyze the methylation reaction of flavonoids more widely than the mutated POMT7. Changes at a few amino acid residues of class II *O*-methyltransferase also have substantial effects on substrate specificity, kinetic property, and regioselectivity [35, 38–40]. These findings indicate that size or shape of the catalytic center of CdFOMT5 plays an important role in determining the broad regioselectivity of CdFOMT5.

### Substrate spectrum of CdFOMT5

To investigate the substrate specificity of CdFOMT5, *O*-methyltransferase activities for naringenin, (-)-epicatechin, equol, and cyanidin were measured (Additional file 2: Figure S4). Recombinant CdFOMT5 exhibited OMT activity for each of these substrates except cyanidin, although detailed structural determinations of the products have not yet been performed. However, we were not able to detect polymethylated products from naringenin, (-)-epicatechin, and equol, even though CdFMOT5 exhibited a broad regioselectivity toward quercetin and monohydroxylated flavone (Fig. 5). The results suggested that CdFMOT5 prefers flavonol (3-hydroxyflavone) to other flavonoid structures. Thus, substrate structure, especially the *C*-ring in flavonoids, may strongly affect the substrate preference, including regioselectivity, of CdFOMT5.

### Bioproduction of polymethylated quercetin with recombinant *E. coli* cells expressing CdFOMT5

**Fig. 6:**
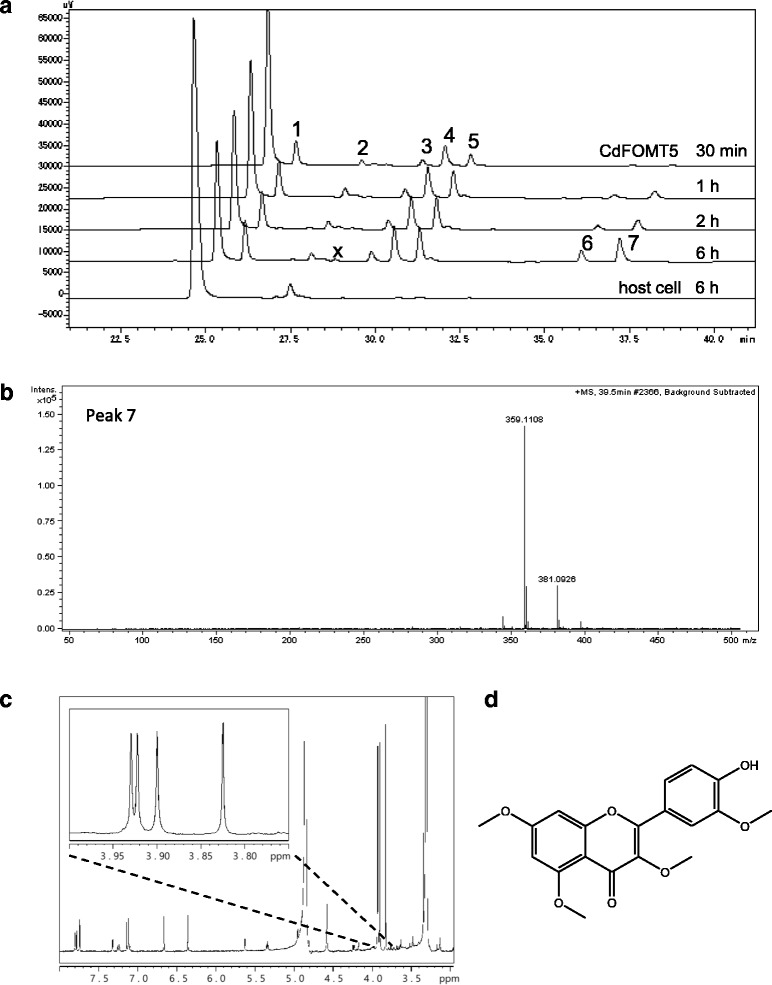
Bioproduction of polymethylated quercetin using recombinant *E. coli* carrying CdFOMT5. **a** The HPLC analysis of the reaction mixture identified five peaks: (1) 3-*O*-methylated quercetin; (2) isorhamnetin (3′-*O*-methylated quercetin); (3) azaleatin/tamarixetin; (4) dimethylated quercetin; (5 and 6) trimethylated quercetin; and (7) 3,5,7,3′-tetramethylated quercetin. Peaks labeled with ‘x’ were not identified as quercetin derivatives. Transformant cells harboring empty vector (pET21b) was used as host cell. **b** Mass spectrometry analysis of purified peak 7 product. **c** NMR spectrum of purified product (peak 7). **d** Predicted structure of 3,3′,5,7-*O*-methylated quercetin

To examine the production of PMF by an *E. coli* biocatalyst expressing CdFOMT5, bioconversion was performed using quercetin as a substrate. In the presence of l-methionine and glucose for regenerating SAM in the reaction mixture, many peaks corresponding to polymethylated quercetins were observed. This agreed with the results of enzymatic reactions of mono-hydroxyflavones. In contrast, negligible product amounts were obtained in the absence of methionine and glucose (data not shown). This result clearly indicates that whole recombinant *E. coli* cells efficiently regenerated SAM using l-methionine and glucose and that the methylation reaction was successfully carried out. Additional peaks that were not expected in the enzymatic reaction were detected (peaks 6 and 7 in Fig. 6a). LC-MS analysis showed that these extra products correspond to tri- and tetra-methylated quercetin (peaks 6 and 7, respectively, in Fig. 6b). Figure 5 shows that CdFOMT5 catalyzes the methylation of the 3-, 5-, and 7-hydroxy groups of monohydroxylated flavones, although it does not catalyzes the 3′ or 4′-monohydroxyflavone (data not shown). Additionally, isorhamnetin (3′-*O*-methylated quercetin) was a product of *E. coli* host cells that lacked CdFOMT5 when used for bioconversion as a control (peak 2 in Fig. 6a). Therefore, we surmised that an unexpected 3′-*O*-methylation reaction occurred in *E. coli* host cells. Thus, the product corresponding to peak 7 was hypothesized to be 3,3′,5,7-tetramethylated quercetin. To verify this, the product of peak 7 was purified and analyzed by H^1^-NMR. As shown in Fig. 6c, four singlet peaks corresponding to the four methoxy groups were detected within a concentration range of 3.8 to 4.0 ppm. This strongly suggests that the product corresponding to peak 7 is 3,3′,5,7-tetramethylated quercetin (Fig. 6d).

## Conclusions

In this study, we successfully obtained five FOMT genes from *C. depressa* and expressed the *CdFOMT5* gene in *E. coli* cells. Recombinant CdFOMT5 demonstrated SAM-dependent *O*-methyltransferase activity for quercetin, and its optimum pH and temperature were 7.0 and 45 °C, respectively. CdFOMT5 exhibited a broad range of not only substrate specificity, but also regioselectivity and catalyzed the methylation of 3-, 5-, 6-, and 7-hydroxyl groups of flavone. Furthermore, quercetin was converted to 3,3′,5,7-tetramethylated quercetin as a sequentially methylated product by the *E. coli* whole cell reaction system. Thus, CdFOMT5 is a useful *O*-methyltransferase possessing a wide range of regioselectivity for flavonoids and likely plays a role in the synthesis of nobiletin in *C. depressa*. Further improvement of the host cell through metabolic engineering and the use of engineered CdFOMT5 would make this bioprocess suitable for producing various PMFs.

## Methods

### Strains and vectors

Fruits of *C. depressa* Hayata (known as shequasar or Taiwan tangerine) were purchased from an agricultural cooperative in Okinawa, Japan to obtain genomic DNA and total RNA. *E. coli* JM109 cells and the cloning vectors pGEM-T Easy (Promega, Madison, WI, USA) and pUC118 were used to clone the *CdFOMT* coding genes. The expression vector pET21b(+) and *E. coli* BL21(DE3) cells were used to express recombinant CdFOMT genes.

### Preparation of genomic DNA and total RNA

Standard techniques were used for DNA manipulation [41]. Genomic DNA and total RNA were obtained from the pericarp of *C. depressa* fruits. Flesh pericarp was ground using a mortar and pestle in the presence of liquid nitrogen. Genomic DNA was extracted by the cetyltrimethylammonium bromide (CTAB) extraction method [42]. Total RNA was prepared using a TRI reagent (Cosmo Bio Co. Ltd., Tokyo, Japan) according to the manufacturer’s protocol. First-strand cDNA was synthesized using a PrimeScript High Fidelity RT-PCR Kit (TaKaRa, Shiga, Japan) with an oligo dT primer, and the products were used as PCR templates to isolate CdFOMT-coding genes.

### Cloning of the CdFOMT-coding genes

To obtain partial fragments of CdFOMT-coding genes, PCR was carried out using genomic DNA as a template. The degenerate primers for amplifying *CdFOMT* fragments were designed from the amino acid sequences of caffeic acid/flavonoid *O*-methyltransferases that are conserved among higher plants. To obtain the full-length CdFOMT-coding genes, TAIL-PCR [43] was carried out based on the deduced partial nucleotide sequences. The full lengths of the cDNA fragment of *CdFOMT* were amplified by PCR using the first-strand cDNA as a template. Amplified fragments were cloned into the pGEM-T Easy vector. All oligonucleotide primers used to clone CdFOMT-coding genes are shown in Additional file 1: Table S1, and all nucleotide sequences were determined using a Capillary DNA Sequencer 3130 (Applied Biosystems, Tokyo, Japan) to perform Sanger DNA sequencing for both strands.

### Nucleotide sequence accession numbers

The nucleotide sequences of the isolated *CdFOMT*s were submitted to the DNA Data Bank of Japan (DDBJ) under the following accession numbers: *CdFOMT1*, LC126056; *CdFOMT3*, LC126057; *CdFOMT4*, LC126058; *CdFOMT5*, LC126059; and *CdFOMT6*, LC126060.

### Expression and purification of recombinant CdFOMT5

Cloned cDNA fragments of CdFOMT-coding genes were excised by *Bam* HI and *Sal* I and purified by agarose gel electrophoresis. Purified DNA fragments were cloned into the expression vector pET21b(+) that had been digested with the same restriction endonucleases. The resulting plasmids were named pET-CdFOMT1 through pET-CdFOMT6, and each of them was introduced into *E. coli* BL21(DE3) cells.

The transformants harboring the *CdFOMT5* gene were grown on LB medium (containing 50 μg/mL ampicillin) to OD_660_ 0.5 at 30 °C with shaking. Isopropyl-β-d-thiogalactopyranoside (IPTG) was added to a final concentration of 0.1 mM, and the cells were incubated for another 24 h at 18 °C to induce the expression of recombinant CdFOMT5. After induction, cells were collected by centrifugation at 33,800 × *g* for 5 min and washed with 50 mM potassium phosphate buffer (KPB; pH 7.0). Cells were harvested by centrifugation and resuspended in cell lysis buffer (50 mM KPB, 200 mM NaCl, 10 % glycerol, 10 mM imidazole, pH 7.2). Disruption of cells was carried out by sonication for 30 min at 4 °C using an Insonator 201 M (Kubota, Tokyo, Japan), and cell debris was centrifuged two times at 33,800 × *g* for 20 min to obtain a clear lysate. The recovered supernatant was applied to Ni-Sepharose chromatography columns (GE Healthcare, Tokyo, Japan; 10 mL bed volume) and washed with 50 mL of the same buffer. Recombinant proteins fused with 6× histidine tags were eluted by the same buffer containing 250 mM imidazole. Collected recombinant CdFOMT5 protein was passed through a desalting column (Econo-Pac PD-10; Bio-Rad Lab., Tokyo, Japan) using 50 mM KPB containing 10 % glycerol. The concentration of the purified recombinant CdFOMT5 was determined using the Bio-Rad Protein Assay kit (Bio-Rad Laboratories, Hercules, CA, USA), and recombinant protein was used for further experiments.

### Determination of the molecular mass of recombinant CdFOMT5

To investigate the molecular mass of native recombinant CdFOMT5, an HPLC analysis was carried out using the Shimadzu LC-10 HPLC system (Shimadzu, Kyoto, Japan) equipped with a Superdex 200 10/300 GL Column (GE Healthcare, Port Washington, NY, USA). First, 100 μL of purified recombinant CdFOMT5 solution was applied and separated using the mobile phase [100 mM KPB containing 200 mM NaCl (pH 7.0), 0.4 mL/min]. Protein absorbance was then monitored at 280 nm, and molecular mass was estimated from the retention times of authentic molecular weight markers (Oriental Yeast Co., Ltd., Tokyo, Japan).

### Enzyme assay

To determine the flavonoid *O*-methyltransferase activity of recombinant CdFOMT5, the purified enzyme was tested for its reaction with quercetin, several hydroxyflavones, and some flavonoids in the presence of SAM as a methyl donor. The reaction mixture consisted of 50 mM KPB (pH 7.0), 500 μM SAM, 100 μM substrate (10 mM in dimethylformamide), and 50 μL of purified CdFOMT5, yielding a total volume of 500 μL. The reaction mixture was incubated at 30 °C for 1 h with shaking (at 1,000 rpm), and the reaction was stopped by addition of 500 μL of methanol. After vigorous mixing, the supernatant was recovered by centrifugation and analyzed by HPLC or LC-mass spectrometry (MS). All enzyme assays were performed in triplicate.

### Bioproduction of polymethylated quercetin by resting-cell reactions

The *E. coli* transformant cells expressing recombinant CdFOMT5 were prepared according to the methods described above. Collected cells from 100-mL cultures were washed and resuspended in a M9 salt medium supplemented with 1 % glucose and 10 mM l-methionine at a final cell concentration of 1 % (*w*/*v*) in a total volume of 0.5 mL. Then, 100 μM quercetin was added to the reaction mixture as a substrate, and the reaction was carried out at 30 °C for appropriate time with shaking. Reaction products were extracted twice with an equal volume of ethyl acetate and dried over anhydrous sodium sulfate, and then the organic solvent was evaporated in a vacuum. Obtained products were dissolved in methanol and analyzed by HPLC or LC-MS.

### HPLC analysis

HPLC analysis of the reaction products was carried out with a Shimadzu LC-10 HPLC system equipped with a Cadenza CD-C18 Column (4.6 mm × 75 mm; Imtakt Corp., Kyoto, Japan). Analytical conditions for quercetin, hydroxyflavones, naringenin, and equol were as follows: eluent A, 0.05 % (*v*/*v*) formic acid; eluent B, acetonitrile; flow rate, 0.5 mL/min; column temperature, 40 °C; sample injection, 5 μL; and a linear gradient program (% eluent B) of 0 to 10 min (15 %), 10 to 25 min (10 to 55 %), 25 to 40 min (55 to 75 %), 40 to 45 min (75 to 15 %), and 45 to 55 min (15 %). The absorbances of products were monitored at 254 nm. The retention time of each compound was as follows: 24.8 min for quercetin; 25.8 min for 3-*O*-methylquercetin; 27.7 min for isorhamnetin (3′-*O*-methylquercetin); 27.8 min for tamarixetin (4′-*O*-methylquercetin); 29.7 min for rhamnetin (7-*O*-methylquercetin)/azaleatin (5-*O*-methylquercetin); 35.2 min for 3-hydroxyflavone; 34.9 min for 3-methoxyflavone; 37.7 min for 5-hydroxyflavone; 30.7 min for 5-methoxyflavone; 29.3 min for 6-hydroxyflavone; 34.3 min for 6-methoxyflavone; 28.2 min for 7-hydroxyflavone; 33.2 min for 7-methoxyflavone; 26.9 min for naringenin; and 27.1 min for equol. The gradient program (% eluent B) for (-)-epicatechin was as follows: 0 to 5 min (10 %); 5 to 22 min (10 to 25 %); 22 to 27 min (25 %); 27 to 30 min (25 to 10 %); and 30 to 35 min (10 %). The retention time of (-)-epicatechin was 12.5 min. The gradient program (% eluent B) for cyanidin was as follows: 0 to 30 min (5 to 100 %); 30 to 35 min (100 %); 35 to 40 min (100 to 5 %); and 40 to 45 min (5 %). The retention time of cyanidin was 8.6 min. Eluents for analysis of (-)-epicatechin and cyanidin were the same as that used for quercetin.

### Mass spectrometry

LC-MS analysis of reaction products was performed using an Agilent 1200 HPLC system (Agilent Technologies, Santa Clara, CA, USA) connected to a Bruker microTOF Mass Spectrometer (Bruker Corporation, Billerica, MA, USA) equipped with an electrospray ionization (ESI) source. The sample (5 μL) was dissolved in methanol and injected into a Cadenza CD-C18 column. Products were separated and eluted under the same HPLC conditions described in HPLC analysis, and positive ion mass spectra were acquired from 50 to 500 m/z.

### NMR analysis of polymethylated quercetin

^1^H-NMR spectra of tetra-*O*-methylated quercetin were recorded on an AV400 spectrometer (Bruker, Rheinstetten, Germany) in DMSO-*d*_6_ (D, 99.9 % with 0.05 % TMS). All signals are expressed as ppm with TMS as the reference. The ^1^H-NMR (400 MHz, DMSO-*d*_6_) spectra of 3,3′,5,7-tetra-*O*-methylated quercetin were as follows;: δ 3.83 (s, ^3^H), 3.90 (s, ^3^H), 3.92 (s, ^3^H), 3.93 (s, ^3^H), 6.36 (d, *J* = 2.4 Hz, ^1^H), 6.66 (d, *J* = 2.0 Hz, ^1^H), 7.13 (d, *J* = 8.8 Hz, ^1^H), 7.74 (d, *J* = 2.0 Hz, ^1^H), 7.79 (dd, *J* = 2.2, 8.6 Hz, ^1^H).

### Chemicals

SAM and 3-*O*-methylated quercetin were purchased from Sigma (St. Louis, MO, USA). All other chemicals were purchased from Wako Pure Chemicals Industries (Osaka, Japan).

## Additional files

Additional file 1: Table S1. Primers for cloning CdFOMT-coding genes from *Citrus depressa*. (*XLSX 14 kb*) Additional file 2: Figure S1. Gene structure of *CdFOMT* sequences from *Citrus depressa*. Exons and introns are expressed as boxes and lines, respectively. Black boxes indicate conserved motifs that are involved in interactions with SAM in plant OMTs. Introns inserted into each gene, in which neighboring amino acid sequences are highly conserved across CdFOMTs, are marked with asterisks. **Figure S2.** (A) Optimum pH and (B) temperature for *O*-methyltransferase activity of recombinant CdFOMT5. Enzyme activity was measured in the following buffers: 50 mM sodium citrate (filled diamonds), 50 mM MES (filled squares), 50 mM phosphate-KOH (filled triangles), 50 mM Tris-HCl (filled circles), and 50 mM glycine-NaOH (multiplication sign). Enzyme activity was measured for 1 h with shaking at various temperatures. **Figure S3.** Mass spectrometry analysis of quercetin products methylated by CdFOMT5. The peak labeled with ‘x’ was not identified as corresponding to flavonoid derivatives. **Figure S4.** HPLC and LC-MS analysis of methylated products of various flavonoids by CdFOMT5. S, substrate control; S + E, enzyme reaction product; P, product peak. For equol, P1 and P2 indicate major and minor monomethylated products, respectively. Peaks labeled with ‘x’ were not identified as flavonoid derivatives. (PPTX 187 kb)

## Abbreviations

FOMT: Flavonoid *O*-methyltransferase
OMT: *O*-methyltransferase
PMF: Polymethoxy flavonoid
SAH: *S*-adenosyl-l-homocysteine
SAM: *S*-adenosyl-l-methionine
