# Supplementary material for: Molecular cloning and characterization of a flavonoid-O-methyltransferase with broad substrate specificity and regioselectivity from Citrus depressa
Source: BMC Plant Biol. 2016 Aug 22;16(1):180. doi: 10.1186/s12870-016-0870-9 (PMC4994406; doi:10.1186/s12870-016-0870-9)
Supplement: Additional file 2: Figure S1. — Gene structure of CdFOMT sequences from Citrus depressa. Exons and introns are expressed as boxes and lines, respectively. Black boxes indicate conserved motifs that are involved in interactions with SAM in plant OMTs. Introns inserted into each gene, in which neighboring amino acid sequences are highly conserved across CdFOMTs, are marked with asterisks. Figure S2. (A) Optimum pH and (B) temperature for O-methyltransferase activity of recombinant CdFOMT5. Enzyme activity was measured in the following buffers: 50 mM sodium citrate (filled diamonds), 50 mM MES (filled squares), 50 mM phosphate-KOH (filled triangles), 50 mM Tris-HCl (filled circles), and 50 mM glycine-NaOH (multiplication sign). Enzyme activity was measured for 1 h with shaking at various temperatures. Figure S3. Mass spectrometry analysis of quercetin products methylated by CdFOMT5. The peak labeled with ‘x’ was not identified as corresponding to flavonoid derivatives. Figure S4. HPLC and LC-MS analysis of methylated products of various flavonoids by CdFOMT5. S, substrate control; S + E, enzyme reaction product; P, product peak. For equol, P1 and P2 indicate major and minor monomethylated products, respectively. Peaks labeled with ‘x’ were not identified as flavonoid derivatives. (PPTX 187 kb) [file 12870_2016_870_MOESM2_ESM.pptx]

## Slide 1
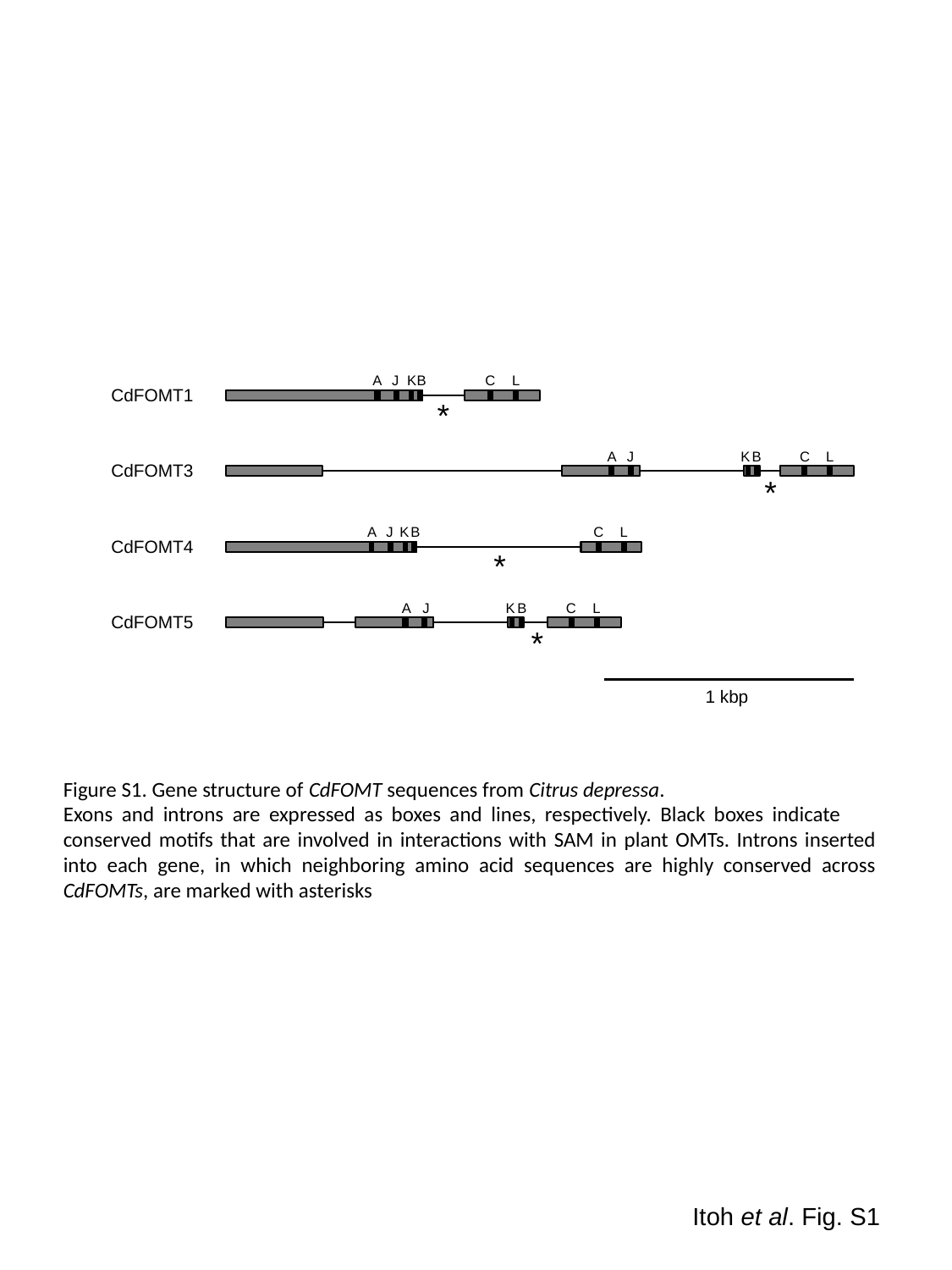

A
J
K
B
C
L
CdFOMT1
A
J
K
B
C
L
CdFOMT3
A
J
K
B
C
L
CdFOMT4
K
B
C
L
A
J
CdFOMT5
1 kbp
*
*
*
*
Figure S1. Gene structure of CdFOMT sequences from Citrus depressa.
Exons and introns are expressed as boxes and lines, respectively. Black boxes indicate　conserved motifs that are involved in interactions with SAM in plant OMTs. Introns inserted into each gene, in which neighboring amino acid sequences are highly conserved across CdFOMTs, are marked with asterisks
Itoh et al. Fig. S1

## Slide 2
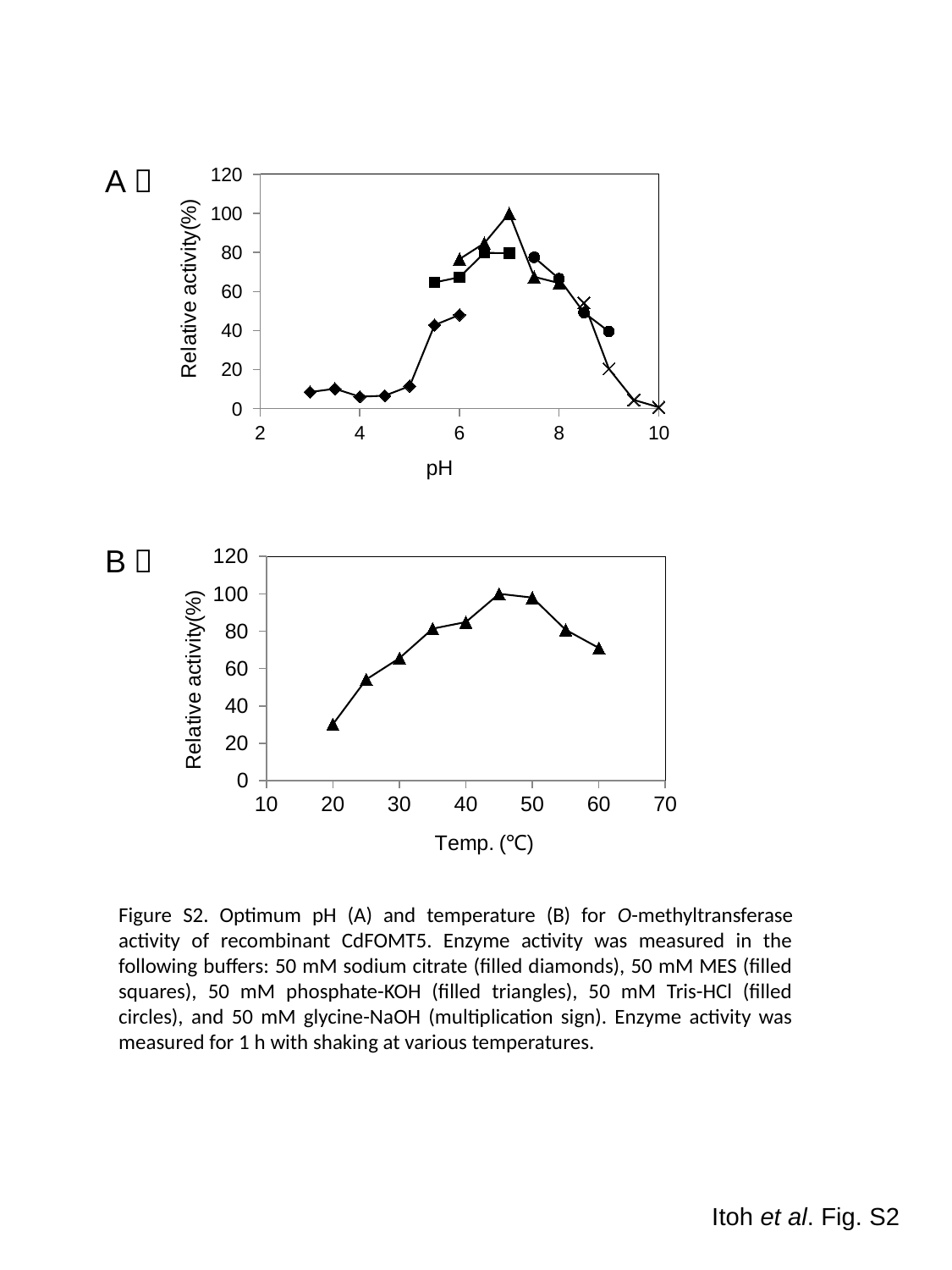

### Chart
| Category | | | | | |
|---|---|---|---|---|---|A）
### Chart
| Category | |
|---|---|B）
Figure S2. Optimum pH (A) and temperature (B) for O-methyltransferase activity of recombinant CdFOMT5. Enzyme activity was measured in the following buffers: 50 mM sodium citrate (filled diamonds), 50 mM MES (filled squares), 50 mM phosphate-KOH (filled triangles), 50 mM Tris-HCl (filled circles), and 50 mM glycine-NaOH (multiplication sign). Enzyme activity was measured for 1 h with shaking at various temperatures.
Itoh et al. Fig. S2

## Slide 3
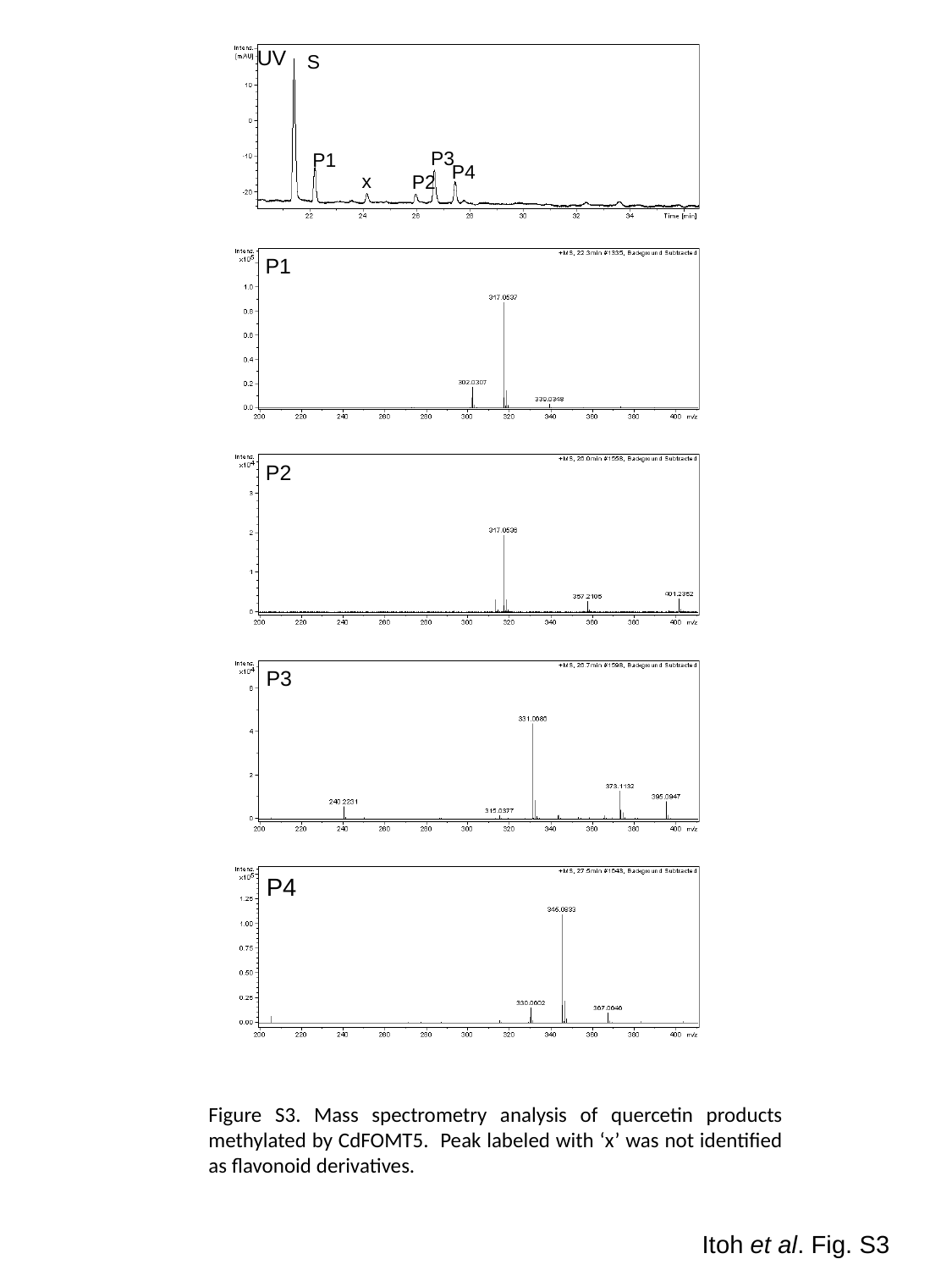

UV
S
P3
P1
P4
x
P2
P1
P2
P3
P4
Figure S3. Mass spectrometry analysis of quercetin products methylated by CdFOMT5. Peak labeled with ‘x’ was not identified as flavonoid derivatives.
Itoh et al. Fig. S3

## Slide 4
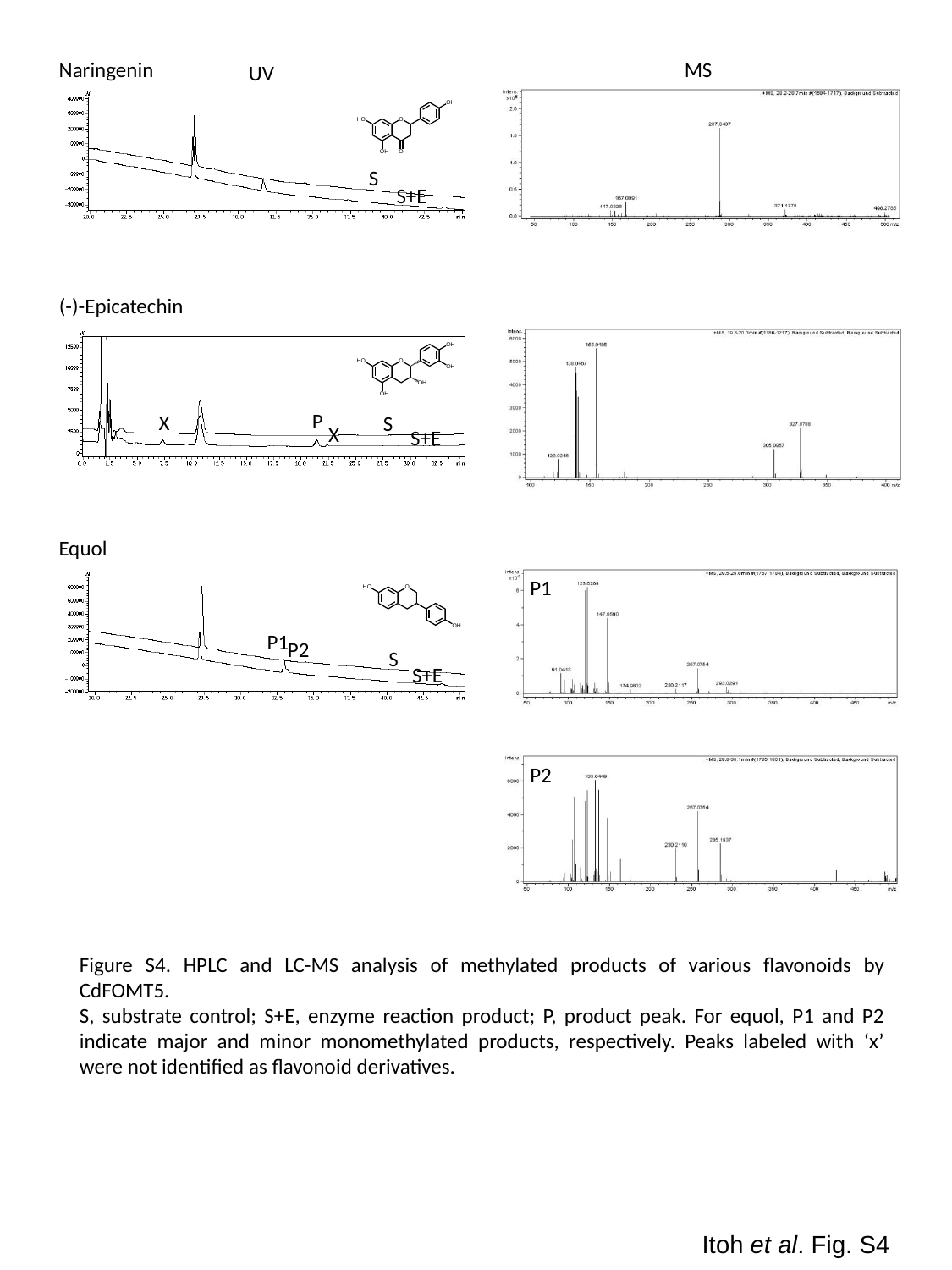

MS
Naringenin
UV
S
S+E
(-)-Epicatechin
P
X
S
X
S+E
Equol
P1
P1
P2
S
S+E
P2
Figure S4. HPLC and LC-MS analysis of methylated products of various flavonoids by CdFOMT5.
S, substrate control; S+E, enzyme reaction product; P, product peak. For equol, P1 and P2 indicate major and minor monomethylated products, respectively. Peaks labeled with ‘x’ were not identified as flavonoid derivatives.
Itoh et al. Fig. S4
